# Effects of a structured exercise program on physical performance and function, quality of life and work ability of physically active breast cancer survivors: A retrospective data analysis

**DOI:** 10.1007/s00508-020-01739-1

**Published:** 2020-09-22

**Authors:** Timothy Hasenöhrl, Stefano Palma, Dominikus F. -X. Huber, Andrej Zdravkovic, Richard Crevenna

**Affiliations:** Department of Physical Medicine, Rehabilitation and Occupational Medicine, Medical University of Vienna, Waehringer Guertel 18–20, 1090 Vienna, Austria

**Keywords:** Dragon boat paddling, Self-help group, Exercise oncology, Exercise is medicine, Breast neoplasm

## Abstract

**Background:**

Purpose of this retrospective data analysis was to depict the effects of a structured off-season conditioning program with breast cancer survivors competing in dragon boat paddling.

**Methods:**

In this study 10 breast cancer survivors (mean age 52.0 ± 5.4 years) who had finished the primary cancer treatment and who were paddlers of the Vienna Pink Dragon self-help group underwent a structured 10-week exercise program as part of their routine supportive treatment. Upper extremity strength, endurance capacity, shoulder flexibility, quality of life and work ability were assessed at baseline and after completion of the exercise program.

**Results:**

Out of 10 patients 8 completed more than 80% of the exercise sessions. A multivariate analysis of variance (MANOVA) of the pooled exercise effects showed a very large effect size (Η^2^ = 0.982); however, the change from baseline to follow-up was non-significant (*p* = 0.363). In the European Organisation for Research and Treatment of Cancer Quality of Life of Cancer Patients Questionnaire (EORTC QLQ-C30) the exercise program led to a significant improvement of body image (*p* = 0.02) and less arm symptoms in the affected arm (*p* = 0.04).

**Conclusion:**

A structured and well-planned exercise intervention program can have a large effect on the physical performance of pretrained breast cancer survivors. Moreover, it can increase the body image and decrease the arm symptoms in this population.

## Introduction

In the early years of research on the effects of exercise in breast cancer survivors (BCS), the first resistance exercise stimulus which was investigated was dragon boat paddling [1]. Back then, it was feared that vigorous and repetitive loading of the upper extremities could negatively affect the status of an existing breast cancer related lymphedema (BCRL) or could provoke the development of a BCRL in the affected arm [2]. Today it is known that this fear was unsubstantiated and strengthening physical activity is not just not harmful but is beneficial in the management of BCRL in BCS [3–5]. Although various exercise stimuli have been investigated regarding feasibility and effects on BCS or BCRL, the historical connection between BCS and the dragon boat sport is still strong today. Dragon boat paddling has become the signature sport for BCS and is undertaken by self-help exercise groups—the so-called Pink Dragons—all over the world [6]. One of those exercise groups, the Vienna Pink Dragons, was founded in 2015 and is under the auspices of the Department of Physical Medicine, Rehabilitation and Occupational Medicine of the Medical University of Vienna, Austria. In this context, the members of the Pink Dragons Vienna were invited to take part in an off-season conditioning program from December 2018 to March 2019. The aim of this uncontrolled clinical trial was to report the pre-post results regarding aerobic capacity, upper extremity strength and mobility, quality of life, and work ability.

## Methods

### Participants

Ten female BCS agreed to participate in the exercise group. Their mean age was 52.0 ± 5.44 years and all participants had finished their primary treatment for breast cancer (BCa). Inclusion criterion was cardiovascular clearance before the exercise program started. Data of patients who attended less than eight of the ten exercise sessions were excluded from the analysis.

Retrospective data analysis was approved by the ethics committee of the Medical University of Vienna, Austria (EK2173/2019).

### Exercise intervention

The exercise intervention was undertaken at the Department of Physical Medicine, Rehabilitation and Occupational Medicine of the Medical University of Vienna, Austria. Ten supervised exercise sessions were provided between December 2018 and March 2019, one session per week except during the holidays. After a short, standardized warm-up procedure for the upper extremities, a circuit training session with two series of resistance exercises using resistance bands and body weight was performed. The choice of resistance exercises was driven by the aim of specific conditioning training for dragon boat paddling: ab-crunches and standing back extensions (body weight) as well as pull-overs, shoulder external and internal rotations, front lifts, torso rotations with resistance bands and an exercise mimicking paddling using a chair, resistance bands and a stick. At the end of each exercise session stretching exercises of the shoulder and upper extremities were performed. Progression was implemented via increasing the exercise time and decreasing the rest time. In the first 2 sessions 40 s exercise alternated with 60 s rest then 5s exercise time was added, and 5s rest time was subtracted for sessions 3 and 4. This progress was repeated until sessions 9 and 10 where the participants exercised 60 s alternating with 40 s rest. Moreover, in the last four sessions a third series of exercises was added. Patients were advised to exert themselves to the limit of correct execution.

### Outcome measures

Before the start of the exercise intervention program and after the last session the participants underwent an assessment battery consisting of a cardiopulmonary exercise testing on an ergometer cycle, strength test of the upper extremities with hand-held dynamometer (Chantillon DFE2-500, AMETEK Inc., Largo, FL, USA) and handgrip strength (JAMAR®, Patterson Medical, Warrenville, IL, USA) as well as a mobility test of the shoulder joints (goniometer). Moreover, the patients filled in questionnaires regarding their quality of life (EORTC QLQ-C30 & BR23) and their work ability (work ability index [7]).

### Statistical analysis

As the performance parameters are a bundle of highly correlated outcomes, performing multiple paired t‑tests would therefore be an inadequate method of analysis, when also considering the small sample size. Instead, performance was analysed as a bundle of outcomes and a multivariate analysis of variance (MANOVA) with two groups (baseline and follow-up) was utilized to report the pooled effect of the exercise intervention. Together with Wilk’s test for significance we reported partial Η^2^ as a measure of effect size. Questionnaires were analyzed category per category with paired Student’s t‑tests.

## Results

Eight of the ten included patients completed the exercise program and attended at least eight of ten exercise sessions. Two patients dropped out of the program, one due to a disease relapse with the necessity for chemotherapy and the other due an event of a chemotherapy-induced late onset cardiotoxicity which needed to be clarified before she could proceed with the exercise program. In this context, she missed too many exercise sessions to be included in the analysis.

### Physical performance

Except for the elbow flexion strength of the affected arm, the mean values of all strength and mobility parameters increased from baseline to follow-up measurements. The results of MANOVA showed no significant change for the pooled performance parameters from baseline to follow-up (*p* = 0.363); however, a very large effect size with a partial η^2^ of 0.982 became apparent (Table 1).

**Table 1:** Physical performance parameters at baseline and follow-up

Physical performance parameter				Baseline mean ± SD	Follow-up mean ± SD	Mean change	
*Handgrip strength (kg)*							
Handgrip affected				30.0 ± 4.8	30.8 ± 5.0	0.8	
Handgrip non-affected				29.0 ± 3.7	31.0 ± 4.5	2.0	
*HHD strength tests shoulder (N)*							
Anteversion affected				128.5 ± 37.2	182.0 ± 9.9	53.5	
Anteversion non-affected				131.6 ± 29.3	191.9 ± 12.6	60.3	
Retroversion affected				161.6 ± 40.5	189.8 ± 39.7	28.2	
Retroversion non-affected				175.9 ± 29.2	210.3 ± 26.3	34.4	
Abduction affected				123.9 ± 20.8	138.0 ± 18.9	14.1	
Abduction non-affected				124.3 ± 26.4	140.9 ± 23.1	16.6	
Adduction affected				172.9 ± 42.4	191.5 ± 39.5	18.6	
Adduction non-affected				187.9 ± 30.6	209.3 ± 26.1	21.4	
External rotation affected				90.9 ± 12.7	102.6 ± 9.1	11.7	
External rotation non-affected				89.8 ± 14.5	98.1 ± 8.8	8.3	
Internal rotation affected				94.0 ± 12.4	106.4 ± 13.9	12.4	
Internal rotation non-affected				97.5 ± 15.5	123.5 ± 15.2	26.0	
*HDD strength tests elbow (N)*							
Flexion affected				169.3 ± 21.0	168.6 ± 18.6	−0.7	
Flexion non-affected				176.5 ± 16.0	182.1 ± 20.3	5.6	
Extension affected				117.3 ± 21.5	133.0 ± 26.3	15.7	
Extension non-affected				113.4 ± 19.8	135.9 ± 32.6	22.5	
*ROM tests shoulder (°)*							
Anteversion affected				170.1 ± 8.3	179.8 ± 9.1	9.7	
Anteversion non-affected				178.0 ± 6.3	182.1 ± 11.0	4.1	
Elevation affected				169.6 ± 9.8	177.4 ± 4.5	7.8	
Elevation non-affected				176.6 ± 5.6	179.5 ± 3.0	2.9	
External rotation affected				88.3 ± 8.6	94.0 ± 9.0	5.7	
External rotation non-affected				89.8 ± 10.1	93.1 ± 9.3	3.3	
*Cardiopulmonary exercise testing*							
Watt max/kg bodyweight				1.7 ± 0.3	1.8 ± 0.4	0.1	
*Multivariate tests*							
*Effect*	–	Value	F	Hypothesis df	Error df	Sig	Partial η^2^
–	Wilks’ lambda	0.018	4.296	13.000	1.000	0.363	0.982

*kg* kilograms, *N* Newtons, ° degrees

### Quality of life

The global health status did not change from baseline to follow-up (*p* = 0.815); however, body image increased (*p* = 0.02) and arm symptoms decreased significantly (*p* = 0.04), both categories representing a beneficial change (Table 2).

**Table 2:** Results of EORTC QLQ-C30 and -BR23 questionnaires (only items with answers of at least 75% of the participants are depicted)

Quality of life parameter	Baseline mean ± SD	Follow-Up mean ± SD	Mean change	Level of significance
*EORTC QLQ-C30*				
Global Health Status	70.8 ± 19.7	72.2 ± 17.7	1.4 ^a^	0.65
Physical functioning	90.0 ± 8.5	91.1 ± 10.0	1.1 ^a^	0.28
Role functioning	70.0 ± 18.9	77.8 ± 25.0	7.8 ^a^	0.40
Emotional functioning	69.2 ± 23.6	64.8 ± 24.9	−4.4 ^b^	0.55
Cognitive functioning	83.3 ± 17.6	81.5 ± 17.6	−1.9 ^b^	0.76
Social functioning	78.3 ± 26.1	90.7 ± 12.1	12.4 ^a^	0.20
Fatigue	31.1 ± 29.1	23.5 ± 21.1	−7.7 ^a^	0.37
Nausea and vomiting	5.0 ± 8.1	1.9 ± 5.6	−3.1 ^a^	0.17
Pain	18.3 ± 21.4	22.2 ± 20.4	3.9 ^b^	0.78
Dyspnea	20.0 ± 23.3	18.5 ± 17.6	−1.5 ^a^	1.00
Insomnia	33.3 ± 38.5	33.3 ± 33.3	0.0	0.73
Appetite loss	3.3 ± 10.5	3.7 ± 11.1	0.4	0.35
Constipation	10.0 ± 22.5	0.0 ± 0.0	−10.0 ^a^	0.35
Diarrhea	6.7 ± 14.1	0.0 ± 0.0	−6.7 ^a^	0.17
Financial difficulties	23.3 ± 35.3	18.5 ± 33.8	−4.8 ^a^	0.35
*EORTC QLQ-BR23*				
Body image	71.7 ± 26.1	90.7 ± 10.6	19.1 ^a^	0.02
Sexual functioning	25.0 ± 21.2	35.2 ± 33.8	10.2 ^a^	0.45
Sexual enjoyment	Not enough data			
Future perspective	40.0 ± 21.1	51.9 ± 29.4	11.9 ^a^	0.51
Systemic therapy side effects	21.0 ± 14.8	15.3 ± 9.8	−5.7 ^a^	0.59
Breast symptoms	7.5 ± 13.9	11.1 ± 10.2	3.6 ^b^	0.59
Arm symptoms	20.0 ± 25.6	8.6 ± 14.5	−11.4 ^a^	0.04
Upset by hair loss	Not enough data			
*Work Ability Index*				
Total score	30.6 ± 11.4	31.0 ± 12.2	0.4	0.88

*EORTC QLQ-C30* European Organisation for Research and Treatment of Cancer Quality of Life of Cancer Patients Questionnaire, *EORTC QLQ-BR23* European Organisation for Research and Treatment of Cancer Quality of Life of Breast Cancer Patients Questionnaire

^a^Indicates beneficial change

^b^Indicates detrimental change

### Work ability

The individual work ability indices ranged from a very low 9.5 to 42.5 points at baseline and from 13.5 to 47 points at follow-up. The mean total score of the work ability index did not change (*p* = 0.838) (Table 2). Individual variability was high in this outcome measure. While one patient reported a substantial increase of 13 points and ascended from “moderate” to “very good” work ability, another patient reported a substantial decrease of 10 points and descended from “good” to “moderate” work ability. Anyhow, this individual deterioration of work ability occurred due to personal reasons and was unrelated to the exercise program.

## Discussion

Physical deconditioning, functional deterioration, sarcopenia and consequently frailty are well documented side effects of various cancer treatments in different cancer entities [8–10]. It has also been well documented that physical exercising is the most effective treatment modality against these side effects [11, 12]. Moreover, exercising does not just positively affect these side effects, but has various systemic effects including the immune system [13] as well as the cardiovascular [14] and metabolic risk situation [15, 16] via helping the patients to maintain a healthier body composition [17]. Better physical fitness can therefore be associated with better side effects management, less comorbidities, higher QoL and even longer survival in cancer patients [9, 18]. Moreover, when considering the processes and timeframe of deconditioning during phases of physical inactivity, increasing physical fitness in any cancer patient—even in already fit ones—will provide each individual with a larger reserve cushion of fitness, functionality, QoL, and their cardiovascular and metabolic risk profile for potential phases of inactivity, for example caused by the necessity for repeated treatment.

In BCS, the side effect profile of the primary treatment is expanded with the potential development of BCRL [19]. About 20% of all BCS develop BCRL which is considered to have a plethora of negative consequences for the patients [20]. Tension and pain, functional deterioration via loss of fine and gross motor skills, stigmatization, and ultimately loss of QoL are well known side effects of BCRL alone [21]. Until the early 2000s BCS were advised to refrain from any vigorous, repetitive physical loading of the affected arm [2]. At that time, it was feared that physical loading of the affected arm could have detrimental effects on an existing BCRL or could trigger a new BCRL.

The shift of paradigm regarding exercise with BCS started with Harris and Niesen-Vertommen (2000) [2]. Due to the lack of a gold standard in terms of the assessment of BCRL, it took 20 years until enough homogeneous data collected with the same assessment method were available for the realization of a thorough meta-analysis regarding the development of the BCRL [5]. This meta-analysis showed a significant beneficial effect of the resistance exercise (RE) on the BCRL when measured with bioimpedance spectroscopy. These findings mark an interim endpoint regarding the question if RE might be detrimental for BCRL [5]; however, bioimpedance spectroscopy is an unreliable method to differentiate between lymphedema volume and muscle mass in BCRL. This is of particular relevance as larger muscle growth rates have been shown in the affected arm of BCS performing a progressive resistance exercise protocol [22]. This means that for future research assessment methods which allow the detailed analysis of arm tissue composition need to be chosen.

As in any study this study has limitations as well. First, it was a retrospective analysis of a routine exercise group, instead of a prospective, controlled study; however, the effect size of this exercise program is large and relevant even without a control group. Second, the small sample size limits the power of the results and increases the risk of a statistical beta error. Being aware of this limitation, we chose an appropriate statistical method with the aim to not analyze single outcome parameters, but the collective effect of similar outcomes.

Historically, in the twentieth century the perception of exercise as a measure in the supportive care of not just cancer but any disease was very critical to nonexistent. The prevalent doctrine went towards resting. Therefore, realizing shift of paradigms is a common experience in the fields of exercise oncology and exercise is medicine [23, 24]. It is currently known that exercise has positive effects on physical capacity, metabolism, chronic inflammation and cardiovascular morbidity and mortality as well as on prevention and mortality in various cancer types, and should therefore be integrated into everybody’s life and also under consideration of the specific risk situation, in the lives of cancer patients [23, 25, 26]. The Pink Dragon movement is a prime example in this respect. It brings BCS together for physical exercise, as well as social interaction and exchange.

The data of this retrospective analysis of our routine exercise group enable the conclusion that a well-planned, performance-oriented exercise program is able to increase physical performance in already trained BCS. This enables this patient population to build up physical fitness reserves for potentially necessary times of physical inactivity.
